# Diallyl Trisulfide Causes Male Infertility with Oligoasthenoteratospermia in *Sitotroga cerealella* through the Ubiquitin–Proteasome Pathway

**DOI:** 10.3390/cells12202507

**Published:** 2023-10-23

**Authors:** Sakhawat Shah, Karam Khamis Elgizawy, Meng-Ya Wu, Hucheng Yao, Wen-Han Yan, Yu Li, Xiao-Ping Wang, Gang Wu, Feng-Lian Yang

**Affiliations:** 1 Hubei Key Laboratory of Insect Resources Utilization and Sustainable Pest Management, College of Plant Science and Technology, Huazhong Agricultural University, Wuhan 430070, China; shahentomology@webmail.hzau.edu.cn (S.S.); wumengya@webmail.hzau.edu.cn (M.-Y.W.); yanwenhan@webmail.hzau.edu.cn (W.-H.Y.); lyct1997@webmail.hzau.edu.cn (Y.L.); xpwang@mail.hzau.edu.cn (X.-P.W.); wugang@mail.hzau.edu.cn (G.W.); 2 Plant Protection Department, Faculty of Agriculture, Benha University, Moshtohor, Toukh 13736, Egypt; karam.elgizawy@fagr.bu.edu.eg; 3 College of Informatics, Huazhong Agricultural University, Wuhan 430070, China; hcyao@webmail.hzau.edu.cn

**Keywords:** spermatogenesis, spermatophore, testis RNA-Seq, apyrene and eupyrene sperm, aggresome

## Abstract

Essential oils extracted from plant sources along with their biologically active components may have negative effects on insects. Diallyl trisulfide (DAT) is an active component of garlic essential oil, and it exhibits multi-targeted activity against many organisms. Previously we reported that DAT induces male infertility and leads to apyrene and eupyrene sperm dysfunction in *Sitotroga cerealella*. In this study, we conducted an analysis of testis-specific RNA-Seq data and identified 449 downregulated genes and 60 upregulated genes in the DAT group compared to the control group. The downregulated genes were significantly enriched in the ubiquitin–proteasome pathway. Furthermore, DAT caused a significant reduction in mRNA expression of proteasome regulatory subunit particles required for ATP-dependent degradation of ubiquitinated proteins as well as decreased the expression profile of proteasome core particles, including β1, β2, and β5. Sperm physiological analysis showed that DAT decreased the chymotrypsin-like activity of the 20S proteasome and formed aggresomes in spermatozoa. Overall, our findings suggest that DAT impairs the testis proteasome, ultimately causing male infertility characterized by oligoasthenoteratospermia due to disruption in sperm proteasome assembly in *S. cerealella*.

## 1. Introduction

From seed germination to product storage, cereal crops, and agricultural food products encounter various forms of damage caused by pests. These damages include, but are not limited to unfavorable climatic conditions, locust plague, and stored product pest infestation. Several insect species cause infestation in stored food products, and they are responsible for the loss of cereal grains [1].

Among the stored product pests, Angoumois grain moth, *Sitotroga cerealella* is considered the common and most destructive pest of cereal grains. Its infestation starts in the standing crop and continues in storage [2]. It damages the stored products such as cereal grains (wheat, rice, and maize), barley, oats, sorghum, millet, flour, meal, stored beans, crackers, pasta, dried pet food, spices, cake mix, chocolate, dried flowers, nuts, seeds, and dried museum [2]. Larvae bore into the grain after hatching and complete their development in grain until the adult stage. The newly emerged adult pushes through the window of the seed coat leaving a small round hole in the crown end of the grain. Considering the behavior of *S. cerealella*, which feeds inside the grains during the larval stage, manipulating the reproductive behavior during the adult stage provides a new mechanism for their control [3]. Similar to other lepidopteran moths, *S. cerealella* produces two morphs of sperm, i.e., Eupyrene and apyrene sperm, which vary in structure and functions. Eupyrene sperm contain a nucleus and DNA, which fertilize eggs while the apyrene sperm lack a nucleus and nuclear DNA and assist the transfer of eupyrene sperm to the female reproductive organ.

The current control strategies for stored product pests depend on synthetic insecticides and chemical fumigants such as organophosphate, aluminum phosphide, and bromomethane [4]. The overuse of these insecticides and fumigants has promoted resistance to several insect species and adverse effects on the environment and off-target species, whereas the use of synthetic fumigants is prohibited because of biosafety concerns [4]. To address the implications of synthetic insecticides, potential alternatives have been developed, such as the use of plant essential oils (EO) and their active components against stored product pests. EO is extracted from several plant leaves, roots, and flowers and it has strong insecticidal activities against stored product pests [5]. Garlic EO is extracted from garlic bulbs, which are composed of organosulfur compounds and possess many potential insecticidal activities against several agricultural product pests [6].

Diallyl trisulfide (DAT) is an active component of garlic EO, which has medicinal applications against cancer and hypertension [7,8]. DAT is considered a source of hydrogen sulfide, which may be used to treat cardiovascular diseases [9]. Apart from these medicinal applications, DAT has insecticidal activities, such as repellent, oviposition inhibitor, and spermicidal activity against *S. cerealella* [10]. Previously we reported that DAT decreased the dichotomous sperm numbers and testis protein contents of *S. cerealella* and impaired the juvenile and ecdysone hormone titers, resulting in lesser oviposition [11]. We further showed that DAT is associated with oligoasthenoteratospermia (OAT), which includes morphological and ultrastructural alterations in dimorphic sperm structure, reduced sperm motility, and arrested spermatogenesis [12]. However, the molecular mechanism of OAT via DAT is unknown.

Ubiquitin proteasome system (UPS) is a major proteolytic pathway in eukaryotes, and 75% of all cellular proteins are UPS substrates. The multistep process of ubiquitination is governed by E1 (ubiquitin-activating enzyme), E2 (ubiquitin-conjugating enzyme), and E3 (ubiquitin ligase) enzymes that sequentially activate, conjugate, and ligate ubiquitin to protein substrate targets. The 26S proteasome is an ATP-dependent proteolytic molecular machine composed of more than 30 different subunits with a molecular mass of over 2400 kDa [13]. The 26S proteasome includes a 20S hollow cylindrical core particle (CP) and a 19S regulatory particle (RP) [14]. The 20S CP is formed by stacking four rings, each composed of seven subunits. Two outer rings incorporate subunits α 1–7, whereas the inner two rings consist of subunits β 1–7 [15]. The 20S CP of the proteasome has multiple enzymatic activities, including chymotrypsin-like, trypsin-like, and peptidyl-glutamyl peptide-hydrolyzing (PGPH) activity. These activities are attributed to the three different types of β-subunits (β1, β2, and β5) that form the inner rings of the 20S CP [16,17]. The 19S RP acts in the recognition, deubiquitylation, unfolding, and translocation of substrates into the 20S CP for proteolysis [18,19]. Mammalian and non-mammalian spermatozoa carry proteasomes that regulate vital fertilization, including sperm penetration of the vitelline layer [20]. Active proteasomal activity tightly regulates several key events of spermatogenesis [20], a complex and highly conserved process with three stages that are regulated by intrinsic signals: spermatogonial mitosis, meiosis of spermatocytes, and spermiogenesis. Dysfunction at any stage leads to defects in sperm maturation and infertility [21]. The UPS plays a crucial role in various aspects of spermatogenesis. It is involved in gonocyte and spermatogonial development, regulates meiotic recombination and progression, modulates spermiogenesis, and participates in acrosome biogenesis [21].

This study aimed to identify and determine the molecular targets of DAT for OAT in *S. cerealella*. RNA extraction and mRNA-Seq experiments were performed to investigate alterations in global gene expression in response to DAT fumigation. This study combined sperm biochemical analysis and transcriptomic profiles to obtain a deeper comprehensive understanding of the molecular mechanism of DAT and the response of *S. cerealella* sperm.

## 2. Materials and Methods

### 2.1. Test Animals and DAT Fumigation

The *S. cerealella* strain associated with wheat was maintained in a laboratory at 28 °C ± 1 °C and 75% ± 5% relative humidity with a photoperiod of 14:10 h (light:dark). This strain was obtained from Wuhan, China, and was used for experimental tests with DAT (purity > 90%, Sigma-Aldrich, Steinheim, Switzerland). Virgin adult male moths were obtained from individual grains containing pupae placed in small glass tubes capped with cotton cloths and held in the laboratory. The pupae were inspected hourly for adult emergence, and then 1-day-old adult male moths were collected for experiments. Ten adult moths were treated by fumigation with DAT at a dose of 0.010 μL/L of air in glass pots (1000 mL, 10 cm diameter × 13 cm height) for 7 h. After treatment, the moths were collected for the subsequent experiments. Three replicates were used for transcriptome and qRT–PCR analyses. Each sample contained more than 80 individuals. Two groups with three replicates from control (CK) and treated (DAT) moths were prepared.

### 2.2. Light Microscopy of Testes and Spermatophore

Briefly, testes of 15 adult moths and spermatophores from the 20 mated females of each group were collected and dissected in phosphate buffer saline (PBS) solution. Samples were isolated and fixed in aqueous Bouin’s solution for 8 h [22]. Samples were dehydrated in a series of increasing concentrations of ethyl alcohol (70%, 80%, 90%, and 100%), cleared in xylene, embedded in histological paraffin, and cut into 3 μm thick sections with a Leica RM 2250 microtome. Sections were stained with hematoxylin and eosin (H&E). Sections were observed under a NIKON Eclipse Ti-SR microscope (Nikon, Tokyo, Japan), and images were captured with a digital camera.

### 2.3. Morphological Analysis of Spermatophore Formation

*S. cerealella* males from both the control and DAT-fumigated groups were paired and mated with virgin females that had emerged within the last 12 h. Then, females were dissected immediately after mating completion, and the morphology of the spermatophore was observed and measured under a microscope (Shunyu, Ningbo, China). Twenty spermatophores were observed for each group, with three biological replicates. Photos were taken by LightTools version 8.6 software.

### 2.4. RNA Extraction, cDNA Library Preparation, and Sequencing

*S. cerealella* adult male moths from the control and DAT-fumigated groups were collected and the testis was dissected in a phosphate-buffered saline (PBS) solution under the stereomicroscope. A rubber pad with holes was used to securely hold the centrifuge tube, which was then placed in a container of liquid nitrogen. This setup allowed the centrifuge tube to float in the liquid nitrogen. After dissecting the testis, it was immediately transferred to the pre-cooled centrifuge tube, which was then placed into the liquid nitrogen for preservation. Total RNA was extracted from the homogenates of the testes using TRIzol reagent (Invitrogen, Carlsbad, CA, USA) according to the manufacturer’s protocol. The concentration and quality of RNA were checked using an Agilent 2100 Bioanalyzer. After DNase I treatment, mRNA was purified from total RNA using magnetic beads with oligo (dT) and fragmented. cDNA was synthesized from mRNA fragments following the protocol for the TruSeq RNA Library Preparation Kit v2 (Illumina, San Diego, CA, USA). After end reparation and adapter ligation, suitable fragments were selected for PCR amplification. Quantification and qualification of the sample library were assessed using an Agilent 2100 Bioanalyzer and ABI StepOnePlus Real-time PCR System. cDNA libraries were then sequenced on an Illumina HiSeq™ 2000 platform.

### 2.5. Transcriptome Assembly and Annotation

After sequencing, raw reads were generated, and low-quality (reads with quality values ≤ 10 were >20%) and adapter-polluted reads with a high content of unknown bases (N) (>5%) were filtered to obtain clean reads. High-quality reads were assembled into contigs using the Trinity program with min_contig_length set to 150 and min_kmer_cov set to 3. The gene family clustering with Tgicl was used to obtain the final unigenes.

Unigenes were aligned by BLAST to the non-redundant nucleotide database (Nt), non-redundant protein database (Nr), Swiss Protein database (Swiss-Prot), Kyoto Encyclopedia of Genes and Genomes (KEGG), and Cluster of Orthologous Groups (COG) to obtain the annotation. With Nr annotation, Blast2GO v2.5.0 was used to obtain the GO annotation, and InterProScan5 (version: v5.11–51.0) was used to obtain the InterPro annotation. The segment unigenesis that best mapped to functional databases in the priority order of Nr, Swiss-Prot, KEGG, and COG was selected as the coding sequence (CDS). We used ESTScan v3.0.2 to predict CDS for unannotated unigenes.

### 2.6. DEG Detection and Functional Enrichment Analysis

After assembly, gene expression levels were evaluated and quantified using fragments per kilobase of the exon model per million mapped reads (FPKM) with RSEM v1.2.12. DEGs between CK and DAT were identified using DESeq2 version 1.30.1 software and the unigene expression. The analysis was based on a negative binomial distribution to perform statistical analysis on raw counts. DEGs were identified on the basis of the fold change ≥1.0 and a *p*-value adjusted to <0.05. DEGs were classified and analyzed using gene ontology (GO) and Kyoto Encyclopedia of Genes and Genomes (KEGG) methods. Online OmicShare Tools (https://www.omicshare.com/tools/) (accessed on 16 Novermber 2022) were used to assess GO enrichment. Fisher’s exact test with *p* < 0.05 was considered enriched. The calculations for the KEGG pathway enrichment analysis were similar.

### 2.7. Quantitative Real-Time PCR (qRT-PCR)

*S. cerealella* proteasome subunit mRNA expression levels were quantified by qRT-PCR on a Bio-Rad iQ2 thermocycler (Bio-Rad, Richmond, CA, USA) to validate the differentially expressed genes (DEG) databases obtained from RNA-sequencing analysis. cDNA was diluted 1:10, and 2 μL was used as input into a 20 μL reaction solution containing 10 μL of SYBR Green Master Mix (Thermo Fisher Scientific, Main St, Miami, USA), 0.8 μL sense and antisense primers, and 6.4 μL of sterilized ultrapure water. Three technical replicates were performed for each biological sample. The reaction conditions were as follows: 95 °C for 30 s, followed by 40 cycles at 95 °C for 5 s and 60 °C for 30 s. A melting curve was obtained at the end of each reaction. Reactions used glyceraldehyde-3-phosphate dehydrogenase as an internal control. Gene-specific primers were designed using Geneious v. 10.1.2. (File S1). A standard curve was produced for primer specificity and efficiency.

### 2.8. Western Blotting

Lysates were prepared using a buffer containing 6.25 mM Tris-HCl, 1% mercaptoethanol, and 10% glycerol for 5 min at 100 °C. Protein concentration was determined using the BCA method (Beyotime, P0012). In general, 20–50 μL of protein was loaded into each well of the SDS-PAGE gel. Volumes of protein samples were made equal by supplementing with 1∗SDS loading buffer (1∗SDS lysis buffer plus 0.002% bromophenol blue). Proteins from immunoprecipitation were dissolved in 1∗SDS loading buffer. Proteins from in vitro reactions were supplemented with 5∗SDS loading buffers (312.5 mM Tris pH 6.8, 10% SDS, 30%glycerol, 25% beta-mercaptoethanol, 0.01% bromophenol blue) to obtain a final SDS concentration of 2%. Electrophoresis was performed using a Mini-PROTEAN Tetra Vertical Electrophoresis Cell system (Bio-Rad). Proteins were then transferred to PVDF membranes (Millipore IPVH00010) using a Mini Trans-blot Module (Bio-Rad). Membranes were blocked with 5% milk and TBST (10 mM Tris pH 7.4, 200 mM NaCl, 0.05% Tween-20) and incubated with a primary antibody overnight at 4 °C. After washing in TBS/T and incubating with a secondary antibody (HRP-conjugated Goat anti-rabbit IgG [H+L], Abclonal AS014), blots were developed using an enhanced chemiluminescence reaction. All Western blot images were acquired by using the ChemiDoc Imaging System (Bio-Rad). The following primary antibodies were used: PSMC1 (Abclonal, A15712), PSMD11 (Abclonal, A15306), ubiquitin (PTM-1106), and GAPDH (Xianzhi biotechnology, Hangzhou, China, AB-P-R 001).

### 2.9. Determination of 26S Proteasome Activation Assay

The 26S proteasome activation assay was determined and analyzed in accordance with the methodology described by [23]. In brief, the testis of control and DAT-fumigated male moths (30 insects/sample) were dissected, lysed in lysis buffer, and centrifuged for 15 min at 15,000 rpm. The supernatant was obtained and the protein concentration in microliter was quantified using the BCA protein kit. The 20S proteasome activity was quantified using the 20S Proteasome Activity Assay Kit (APT280; Chemi-Con, USA). The assay was incubated for 2 h (scan every 30 min) at 37 °C in a 96-well plate. Fluorescence intensity was measured using a 380/460 nm filter set equipped with a fluorometer. The experiment was performed in triplicate with biological replicates, and 90 insects from each group were used.

### 2.10. Detection of Aggresomes

Aggresome detection assays were performed using the Proteostat^®^ Aggresome detection kit (Enzo Life Sciences, Farmingdale NY, USA). The procedure was followed as described for Drosophila brain [24] and through personal communication with the first author. Briefly, the dissected testis from the control and experimental groups was fixed in 4% formaldehyde and permeabilized with 0.5% Triton X-100. The slides were then stained with aggresome detection reagent for aggresome and DAPI for nuclear DNA staining. The aggresomes were detected under an Olympus FV1000 laser confocal microscope.

### 2.11. Statistical Analysis

The data were analyzed using a one-way analysis of variance (ANOVA) and Student’s *t*-test. All the figures were generated using GraphPad Prism 8 (GraphPad Software Inc., San Diego, CA, USA). The data are presented as the mean  ±  standard deviation (mean  ±  SD). Statistical tests used to determine significance are indicated in the figure legends.

## 3. Results

### 3.1. DAT Caused Disruption in Testis and Sperm Bundle Morphology of S. cerealella

**Figure 1:**
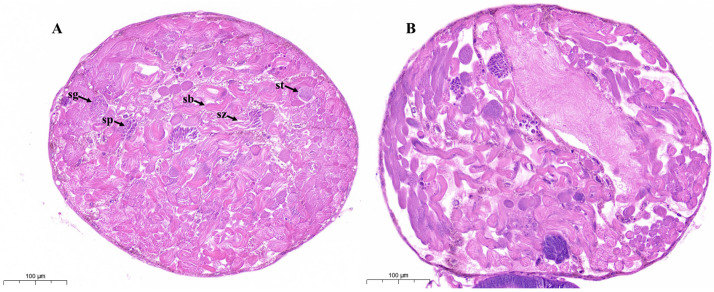
Light microscopy of adult *S. cerealella* testes. (**A**) Representative images of light microscope of control adults moth testes. The testes were collected from 15 virgin adults and stained with hematoxylin and eosin. The section indicates spermatogonia (sg), spermatocytes (sp), spermatids (st), spermatozoa (sz), normal sperm bundles (sb), and cyst distribution. (**B**) Representative images of histological sections from adult testes fumigated with DAT indicate disruption in testes morphology and improper and irregular distribution of spermatozoa. Scale bars, 100 μm.

An adult male *S. cerealella* possesses a single fused dark brown, round testis (Figure 1). The control testis showed normal morphology and distribution of cysts and sperm bundles (Figure 1A). The H&E staining of control testis revealed the growth, maturation, and differentiation of germ cells. Spermatozoa are loosely bundled and exhibit a filamentous spiral arrangement (Figure 1A).

After DAT exposure, the shape and morphology of adult testes were remarkably altered and displayed notable cellular stress. DAT substantially disrupted the distribution and organization of spermatocytes, spermatids, cysts, and sperm bundles and affected sperm numbers. Histological sections contain empty spaces and possess an unbalanced and disorganized distribution of spermatozoa (Figure 1B). Cytoplasmic vacuoles were seen along with many spaces between sperm bundles and cysts and significant tissue reduction. The negative effects of DAT on adult moth testes, as described above, suggest damage to dimorphic spermatogenesis.

### 3.2. DAT Induces Abnormal Spermatophore Formation in S. cerealella

**Figure 2:**
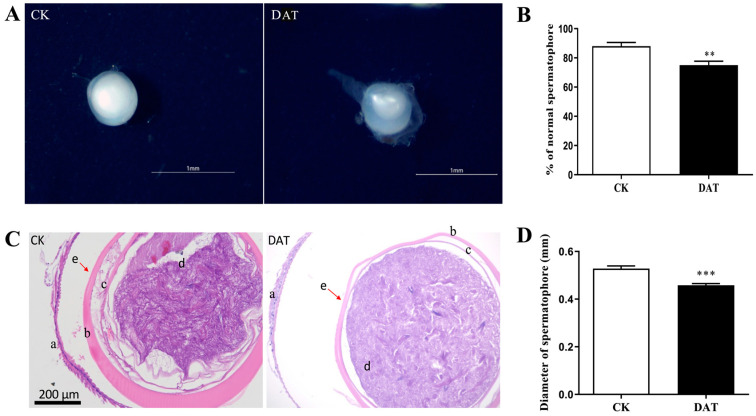
Morphological and histological analysis of *S. cerealella* spermatophore. (**A**) The morphology of spermatophores of control (CK) and female mated with DAT-fumigated males (DAT). (**B**) The rate of the normal spermatophore quantification. ** *p* < 0.01, (*t*-test) (**C**) Histological sections of spermatophore of control (CK) and female mated with DAT-fumigated males (DAT). a: spermatophore capsule wall; b: exterior envelope; c: interior envelope; d: ejaculatory contents (sperm, seminal fluids, z and nutrients); e: change in the outer layer of the spermatophore. (**D**) The diameter measurement of the spermatophore of *S. cerealella*. *** *p* < 0.001, (*t*-test).

To investigate the structural characteristics of spermatophores, which function as reservoirs for sperm and ejaculatory substances, female *S. cerealella* were dissected following successful mating. The results demonstrated that females mated with control males exhibited fully developed spermatophores, which were characterized by their spherical shape and milky-white content (Figure 2A, CK). In contrast, females mated with DAT-fumigated males displayed abnormal spermatophores with reduced contents (Figure 2A, DAT). A quantitative assessment of normal spermatophore formation revealed that the experimental group displayed a reduced rate of normal spermatophore formation (75 ± 2.70%) compared to the control group (88 ± 2.60%), (*p* = 0.008, df = 8, F = 0.031) (Figure 2B).

Histological analysis of the spermatophores demonstrated distinct morphological differences between females mated with control males and those mated with DAT-fumigated males. The sperm bundles within the spermatophores of the control group were densely packed, abundant, and displayed a deep purple color, resembling thread-like structures. However, females that mated with DAT-fumigated males showed a reduced quantity of sperm bundles within the spermatophores (Figure 2C). Additionally, a significant change was observed in the diameter of the spermatophores between females mated with fumigated males (0.46 ± 0.0073 mm) and the control group (0.52 ± 0.01 mm), with the DAT-fumigated group resulting in a smaller diameter of spermatophores compared to the control group (*p* = 0.000, df = 126, F = 0.69) (Figure 2D). These findings collectively indicate that DAT fumigation significantly affects the quantity of sperm ejaculated by males, leading to abnormal spermatophore formation.

### 3.3. RNA-Seq Revealed the Global Downregulation of Gene Expression in Response to DAT Fumigation

**Figure 3:**
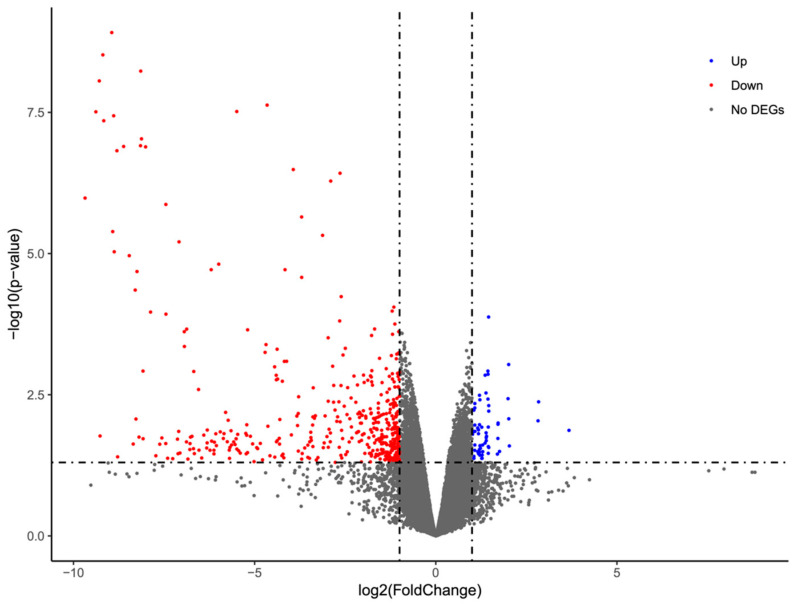
Testis RNA-Seq identification of differentially expressed genes (DEGs) between the control (CK) and DAT-fumigated moths. Volcano plot of the comparison between CK and DAT-fumigated moth testis. The vertical dash lines indicate log2(fold change) = 2, and the horizontal line indicates *p*-value < 0.05. Upregulated and downregulated DEGs were represented in blue and red, respectively.

The transcriptome analysis of control and DAT-fumigated moths with three biological replicates was conducted. A total of 83,788 transcripts were detected with an average FPKM value greater than 1.0 in at least one experimental group. Pairwise differential gene expression analysis was performed between the control and treatment groups to identify DEGs. The number of upregulated DEGs and downregulated DEGs was 60 and 449, respectively (Figure 3, File S2). Notably, the downregulated DEGs had significantly greater fold change than the upregulated genes (Figure 3). Collectively, the transcriptome pattern indicated global downregulation of gene expression in response to DAT fumigation.

### 3.4. GO and KEGG Downregulated Genes Enriched in the Ubiquitin Proteasome System (UPS)

**Figure 4:**
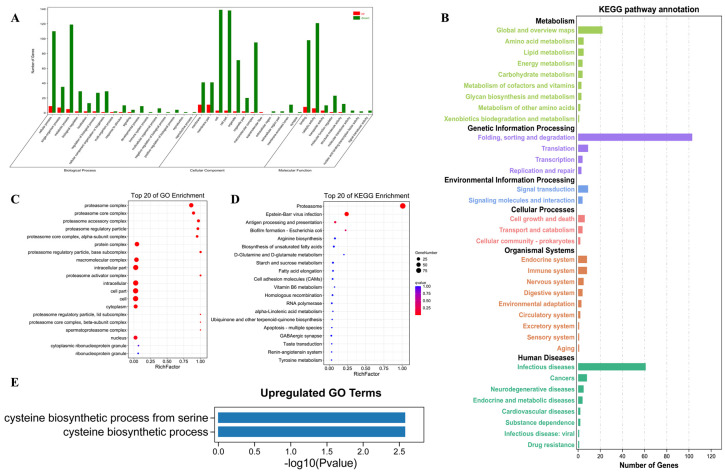
Analysis of differentially expressed gene profiles. (**A**) GO terms and pathway enrichment analyses of differentially expressed genes (DEGs) in CK and DAT-fumigated testis of adult *S. cerealella*. (**B**) Kyoto Encyclopedia of Genes and Genomes classification for DAT samples compared with CK. Enriched functional GO terms (**C**) and KEGG (**D**) for downregulated genes in DAT samples compared with CK. (**E**) Enriched functional GO terms for upregulated genes in DAT samples compared with CK. (**C**,**D**) The size and color of the bubbles represent the gene number and q-value, respectively.

The GO functions of the identified DE genes were classified into cellular components, molecular functions, and biological processes (Figure 4A). The KEGG metabolic pathways of DEGs were classified into six subcategories, namely, metabolism genetic information processing, environmental information processing, cellular processes, organismal systems, and human diseases (Figure 4B).

GO enrichment analysis was performed to identify the altered biological functions and pathways of the DEGs between the control and experimental groups. A total of 18 GO terms were significantly enriched for the downregulated genes in the DAT samples (Figure 4C). The downregulated terms include proteasome complex; proteasome core complex; proteasome accessory complex; proteasome regulatory particle; proteasome alpha and beta subunits; proteasome lid and base subunits; and proteasome activator complex. In exploring the molecular function and metabolic pathway activity alterations after DAT fumigation, KEGG reference pathways were examined with regard to downregulated DEGs downregulated after DAT fumigation. KEGG analysis also revealed significant functional terms related to proteasome, viral infection, and antigen processing and presentation among downregulated genes (Figure 4D). Few upregulated DEGs were identified, and they were mostly associated with cysteine biosynthesis (Figure 4E). Collectively, the RNA-Seq data revealed that DAT fumigation significantly alters the ubiquitin–proteasome pathway by downregulation essential proteins involved in ubiquitination and degradation.

### 3.5. Validation of RNA-Seq Data and the Effects of DAT on the Expression of Proteasome Subunits

**Figure 5:**
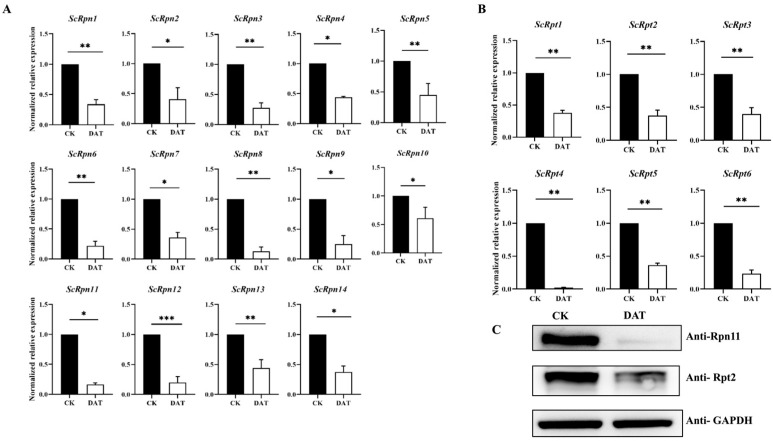
Normalized relative expression of *ScRpns* and *ScRpts*. (**A**,**B**). mRNA expression analysis of *ScRpns* and *ScRpts* mRNA from control and DAT-fumigated adult moths. Pupae of *S. cerealella* were observed, and newly emerged male adults were placed in separate glass bottles. After 12 h, the moths were fumigated with DAT for 7 h in a 1-L glass Jar, and control moths were kept with air. RNA was extracted from control and DAT-fumigated moths testis, and the mRNA expression level of each *ScRpns* and *ScRpts* was analyzed by qRT-PCR as described in the Methods section. The mRNA expression level of each *ScRpns* and *ScRpts* was normalized with GAPDH. Error bars depict the standard deviation (SD) of the mean of three independent replicates. (**C**). Western blot analysis of *Rpn6* and *Rpt2* protein levels in adult *S. cerealella* of control and DAT-fumigated adult moths. GAPDH was used as an internal control. Treatment was compared with their respective control. (Student’s *t*-test) * *p* < 0.05, ** *p* < 0.01, *** *p* < 0.001.

Functional enrichment analysis identified DEGs enriched primarily in the proteasome, proteasome subunit complex, proteasome regulatory particles, and proteasome assembly. A total of 14 genes encoding non-ATPase regulatory subunits (*Rpns*) and six ATPase regulatory subunits (*Rpts*) were found in the testis RNA-Seq data. The mRNA expression of all subunits was downregulated after DAT treatment. We used qRT-PCR to quantify the mRNA expression of *ScRpns* and *ScRpts* in control and DAT-treated insects to confirm RNA-Seq data and evaluate the expression of proteasome subunits in adult moths. The mRNA expression levels of *ScRpns* and *ScRpts* were downregulated (Figure 5A,B), which indicates the integrity of the RNA-Seq data. Moreover, representative proteins, namely, *Rpn11* (known as *Rpn6* in mammals and *Drosophila*) and *Rpt2*, were significantly downregulated after DAT exposure, based on Western blot analysis (Figure 5C, File S3).

### 3.6. DAT Inhibited the Expression of the Proteasome Catalytic Core Complex

**Figure 6:**
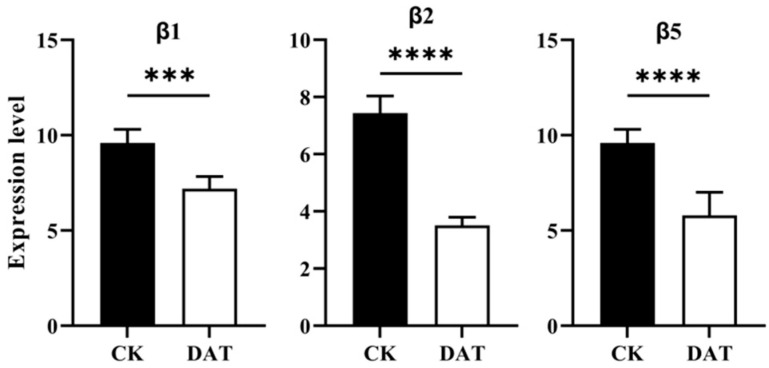
mRNA expression of proteasome core complex particles. Expression level of β1, β2, and β5 mRNA in adult moths. DAT significantly decreases the expression level of these genes *** *p* < 0.001. Data are reported as mean ± SEM ****p < 0.0001.

Because DAT inhibited the mRNA expression level of genes involved in 26S proteasome machinery, the expression profile of proteasome catalytic core particles, as well as β1, β2, and β5 of the proteasome, was checked, which show glutamyl peptide hydrolyzing activity (PGPH), trypsin (Try-L), and chymotrypsin-like (ChT-L) activity. The mRNA expression of β1, β2, and β5 was significantly reduced by DAT fumigation (Figure 6). The inhibition of mRNA expression levels of β1, β2, and β5 indicates that DAT likely inhibits proteasome PGPH, Try-L, and ChT-L activities.

### 3.7. DAT Inhibited 26S Proteasome Activity and Formed Aggresomal Proteins in the Testes

**Figure 7:**
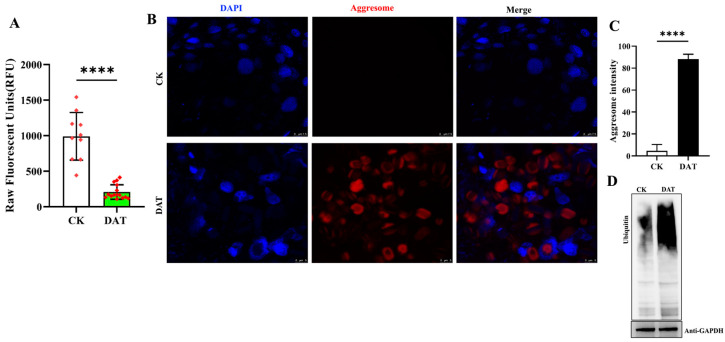
Proteasome activity assay and aggresomal formation. (**A**) Chymotrypsin-like activity of 20S proteasome activity in control and DAT-fumigated adult moth testis. Proteasome activity was monitored with specific fluorescent substrate. Relative proteasome activity represented the percentage of fluorescence compared with the control (*p* < 0.0001). (**B**,**C**) DAT promotes the accumulation of protein aggresomes in the testis. Representative images of 4′,6-diamidino-2-phenylindole (DAPI) staining (blue), ProteoStat aggresome detection reagent (Red), and a marker of protein aggregation in testis are shown. The number of cells with aggresomes in merged images of DAT-fumigated samples compared with control. Scale bars, CK = 7.5 μm, DAT = 5 μm. (**D**) Western blot analysis of ubiquitin in adult *S. cerealella* of control and DAT-fumigated adult moth testis. GAPDH was used as an internal control. Data are reported as mean ± SEM (**** *p* < 0.0001).

In addition, the effects of DAT on proteasome activity were determined, and the ChT-L activity was measured in the testis of control and DAT-fumigated moths. Testes obtained from control and DAT-fumigated adult moths were isolated using the proteasome activity fluorescent peptide substrate (Suc-LLVY-AMC), and homogenates were normalized to total protein levels. The assay indicated a significant reduction (*p* < 0.0001) in proteasome activity in the testis of DAT-fumigated moths compared with the control (Figure 7A). The inhibition of proteasome activity causes aggresomal formation, which is a cellular structure containing ubiquitinated and misfolded proteins. The proteasome inhibition was further analyzed by fluorescence microscopy based on the aggresomal formation. The testes isolated from control and DAT-fumigated moths were stained with ProtesStat dye specific for aggresomes. The results revealed that the inhibition of proteasome activity through DAT resulted in aggresomal formation compared with the control (Figure 7B,C). A higher density of aggregates was found in the cytoplasm compared with the control group. An increase in ubiquitinated protein level was observed in DAT-fumigated testis samples compared with the control, because of the decrease in proteasomal activity in the testis as detected by the ubiquitin antibody (Figure 7D). Collectively, these results demonstrate that DAT serves as a potent proteasome inhibitor and inhibits Try-L and CHT-L activities in the testis of moths, which leads to unbalanced protein degradation, thereby causing aggresomal formation and increased ubiquitinated protein levels.

## 4. Discussion

Testis RNA-Seq analysis between control and DAT fumigation revealed 449 downregulated and 60 upregulated genes. GO and KEGG enrichment analyses of downregulated genes revealed the enrichment of genes in the ubiquitin–proteasome pathway (UPS). The *Bactrocera dorsalis* testis transcriptome reveals genes related to UPS that are involved in spermatogenesis and reproduction [25]. These genes have a role in testis development and spermatogenesis. Similarly, the analysis of the testis transcriptome of *Drosophila melanogaster* reveals the presence of ubiquitin–proteasome system pathway genes and their function in late spermatogenesis and spermiogenesis [26]. Furthermore, our expression assay and Western blotting concluded that DAT significantly decreased the mRNA expression and protein level of proteasome regulatory particles, respectively. Studies indicate that the proteasome regulatory particles are required in *Drosophila* for sperm individualization and maturation during spermatogenesis [27]. Similarly, the knockdown of proteasome regulatory particles in brown planthopper *Nilapervata lugens* impaired ovarian development and oocyte maturation, resulting in reduced fecundity [28]. The testis of DAT-fumigated adult *S. cerealella* showed abnormal morphology and spermatogenesis defects compared with control while the expression profile of the catalytic core particles, β1, β2, and β5 of the proteasome assembly was also significantly reduced by DAT fumigation. Previous studies revealed that proteasome complexes constitute Lepidopteran-specific pathways associated with apyrene sperm activation and these proteins are involved in the degradation of the extracellular fibrous matrix in which eupyrene sperm are bundled [29,30]. We hypothesize that DAT targets the testis proteasome system, leading to apyrene and eupyrene sperm dysfunction in *S. cerealella*.

The successful fertilization process in *S. cerealella* relies on the formation of a normal spermatophore, which facilitates the transfer of mating contents from the male to the female storage organ, the spermatheca. This transfer includes seminal fluid, sperm, accessory gland contents, and nutrients. Some studies have suggested that certain components within the spermatophore also influence the development of the ovaries, thereby enhancing female reproductive capacity; thus, the normal formation of spermatophores holds a pivotal role in ensuring successful fertilization [31].

Histological analysis and quantification of sperm content show that spermatophores generated by females mated with DAT-fumigated males exhibit significant abnormalities and reduced size. Statistical analysis further demonstrates a significant decrease in the rate of normal spermatophore formation among DAT-fumigated male moths compared to the control group. This observation indicates that DAT leads to a reduction in the transfer of mating contents from male to female moth bursa copulatrix during the mating process, with potential implications for fertilization. Similarly, when the triglyceride lipase gene was knocked down in *S. cerealella*, it resulted in the formation of abnormal spermatophores and a significant reduction in sperm quantity within the spermatophore [32]. Triglyceride lipase is known to play a crucial role in lipid metabolism and energy balance, and it has been previously reported that DAT leads to decreased lipid content and disrupts the energy metabolism of adult *S. cerealella* moths [33]. Additionally, our previous study indicated a significant reduction in the motility of both apyrene and eupyrene sperm following DAT fumigation [12], which is essential for sperm transfer from the storage organ to the fertilization site, whereas the osmotic female processes are also essential for filling the spermatheca in certain insect species [34]. The abnormal spermatophore formation may be linked to the malfunction of proteasome assembly and related proteins in the testis as the transcriptome and proteome analyses of the spermatophore revealed enrichment of expression in proteins associated with the ubiquitin–proteasome pathway [35].

Our physiological analysis revealed that DAT decreases the chymotrypsin-like (ChT-L) activity of the proteasome in adult moths using the fluorescent peptide substrate (Suc-LLVY-AMC), which leads to an increased level of ubiquitinated proteins. Similar results were revealed when the chymotrypsin-like activity of the proteasome was selectively inhibited by green tea polyphenols [36]. Several other natural products have been screened for possible proteasome inhibition, such as marchantin, which is bound to the β1 and β5 proteasome core particles and inhibits the proteasome activity in vitro and in intracellular systems [37]. Similarly, the organosulfur compound, Ajoene, derived from garlic Ajoene, is reported to inhibit trypsin-like activity of the 20S proteasome in vitro [38]. Recently, indirubin, an active component of traditional Chinese medicine, was reported to inhibit proteasome activity [39]. In another study, the decreased proteasome level resulting from the knockout of the alpha subunit PSMA8 increased the levels of ubiquitinated proteins in the testis [40]. The fluorescence microscopy analysis of the testis from the experimental group indicated aggresomal structures compared with the control. Cells that suffer from abnormal proteasomal inhibition cause aggresomal structure formation as unwanted proteins and other cellular debris remain in the cell rather than being degraded via proteasomal machinery.

Sperm biochemical analysis for the proteasome activity and aggresomal formation along with RNA-Seq findings revealed that testis proteasomal proteins are the primary target of DAT. However, the mechanism by which DAT inhibits the proteasome pathway remains unknown. Different natural products and synthetic proteasome inhibitors are inhibited by various pathways. Selective proteasome inhibitors exhibit specialized functions, such as targeting different E3 ligases, as each E3 ligase is specific to a particular small set of proteins. We found that DAT inhibited the expression level of the RING finger E3 ligase Seven-in-Absentia (SINA) mammalian homolog *Siah1* in adult moths compared with controls. We hypothesize that DAT may inhibit ubiquitination and 26S proteasome-mediated degradation through the RING finger E3 ligase *SINA*. The knockdown of E3 *SINA* in mice leads to sterility and blocks meiosis I during spermatogenesis [41].

## 5. Conclusions

DAT impairs normal proteasomal regulation and activity through complex mechanisms in male moths, thereby disrupting the apyrene and eupyrene spermatogenesis. Significant downregulation of proteasome 20S CP and 19S RP may compromise sperm metabolism and delay spermatogenesis. Conversely, several other proteasomes are associated with sperm proteins and genes involved in sperm capacitation, motility, and sperm-egg fusion in mammalian and non-mammalian species [42]. Therefore, further exploration of testicular tissues is necessary to comprehensively understand 26S proteasome proteins.

## Data Availability

Not applicable.

## Supplementary Materials

The following supporting information can be downloaded at: https://www.mdpi.com/article/10.3390/cells12202507/s1, File S1: Primers set; File S2: DEGs; File S3: WB quantification.
